# Isolation of Cellulolytic *Bacillus subtilis* Strains from Agricultural Environments

**DOI:** 10.5402/2012/650563

**Published:** 2012-02-29

**Authors:** Yu-Kyoung Kim, Shin-Chan Lee, Young-Yun Cho, Hyun-Jeong Oh, Young Hwan Ko

**Affiliations:** ^1^Division of Sustainable Agriculture Research, Jeju Agricultural Research and Extension Services, Jeju-do 697-828, Republic of Korea; ^2^Research Division, Bio-Agr Co. Ltd., Jeju-shi, Jeju-do 690-022, Republic of Korea; ^3^Department of Food Bioengineering, Jeju National University, Jeju-do 690-756, Republic of Korea

## Abstract

The bioconversion of cellulose and hemicellulose to soluble sugars is important for global stabilization and a sustainable human society. Here, hundreds of cellulolytic bacteria were screened and isolated from soil, compost, and animal waste slurry in Jeju Island, South Korea. Among the isolates, three strains, SL9-9, C5-16, and S52-2, showing higher potential for practical uses were purified on carboxymethyl cellulose (CMC) agar plates and identified as *Bacillus subtilis* strains by morphological, physiological, and biochemical characterization and 16S rRNA gene analysis. The production patterns of cellulose or hemicellulose-degrading enzymes were investigated during cell culture. All three isolated strains produced CMCase, Avicelase, *β*-glucosidase, and xylanase enzymes, which suggested synergic cellulolytic systems in *Bacillus subtilis*. The enzymes showing CMCase, Avicelase, and xylanase activities existed in cell-free culture supernatant, meanwhile *β*-glucosidase activity was detected in cell debris suggesting that three of the enzymes, including CMCase, Avicelase, and xylanase, were extracellular, and *β*-glucosidase was cell membrane bound. The three isolates, SL9-9, C5-16, and S52-2, were not the same strains, presenting slight differences in biochemical characteristics, 16S rRNA gene sequences, and cellulolytic enzyme activities.

## 1. Introduction

The bioconversion of cellulose to soluble sugars and glucose is catalyzed by a group of enzymes called cellulases that are produced by microorganisms [1].

These cellulolytic microorganisms play an important role in the biosphere by recycling cellulose, the most abundant and renewable biopolymer on Earth. The demand for microbial cellulases and related enzymes is growing more rapidly than ever before [2].

Fungal cellulases are produced in large amounts, which include all the components of a multienzyme system with different specificities and modes of action, that is, endoglucanases (or CMCase), exoglucanases (cellobiohydrolase), and *β*-glucosidases (or cellobiases), acting in synergy for the complete hydrolysis of cellulose [3–6]. Synergy between cellulase components during the hydrolysis of cellulose in *Trichoderma viride* was first demonstrated by Giligan and Reese [7].

Synergic multienzyme systems are also expected in bacterial cellulase complexes. Cellulolytic bacteria include aerobes such as *Pseudomonas* and *Actinomyces,* facultative anaerobes such as *Bacillus* and *Cellulomonas*, and strict anaerobes such as *Clostridium*. Most of these bacteria produce mainly endoglucanases [8]. A variety of *Bacillus* species secrete cellulases, including strains of *B. cereus *[9], *B. subtilis* [10], *B. licheniformis* [11], *Bacillus* sp. KSM-330 [12], and alkaliphilic *Bacillus *[13]. In addition, a fairly common observation has been that bacilli lack a complete cellulase system, with primary activity being on carboxymethyl cellulose (CMCase, endoglucanase), and which do not hydrolyze crystalline cellulose [10]. However, in contrast, there are reports of certain *Bacillus* endoglucanases (CMCase) that have shown detectable activity on microcrystalline cellulose [14, 15].

We isolated cellulase-producing *Bacillus subtilis* strains from soil, compost, and animal waste slurry and studied their cellulolytic enzymes. This paper reports the occurrence of these cellulolytic enzymes from *Bacillus subtilis* strains isolated from different habitats. According to the results, the strains possess microcrystalline cellulose-hydrolytic activity, cell-bound *β*-glucosidases, and hemicellulases in addition to endoglucanases.

## 2. Materials and Methods

### 2.1. Bacterial Strains

Three strains, *Bacillus licheniformis* KACC10476, *B. pumilus* KACC10917, and *B. subtilis* KACC10111, were obtained from Korean Agricultural Culture Collection (KACC, Rural Development Administration, Korea). Four strains, *B. amylolicheniformis* KCTC2105, *B. licheniformis* KCTC3045, *B. pumilus* KCTC3348, and *B. subtilis* KCTC3560, were obtained from Korean Collection for Type Cultures (KCTC, Korea Research Institute of Bioscience and Bioengineering, Korea). Three bacterial isolates were acquired in this study and deposited in the KACC under registration number KACC91232P for *Bacillus subtilis* SL9-9, KACC91229P for *Bacillus subtilis* C5-16, and KACC91233P for *Bacillus subtilis* S52-2.

### 2.2. Isolation of Bacteria Producing Cellulases

A total of 176 samples were collected from soil, compost, and animal waste slurry on Jeju Island, South Korea, and were screened for cellulolytic bacteria. The samples were stored at 4°C in the dark until use. After appropriate dilutions with sterile water, 1 mL each of the sample dilutions was spread onto carboxymethyl cellulose (CMC) agar plates that consisted of CMC, 10.0; yeast extract, 1.0; (NH_4_)_2_SO_4, _2.5; K_2_HPO_4_ ·3H_2_O, 0.25; NaCl, 0.1; MgSO_4_ ·7H_2_O, 0.125; FeSO4·7H_2_O, 0.0025; MnSO4·4H_2_O, 0.025; agar, 10(g/L, each), and then the plates were incubated at 28°C for 2 days. The incubation temperature focused on mesophiles. The initial medium pH was adjusted to 7.0 if not specified. A few bacterial colonies were harvested and transferred to fresh CMC agar plates containing trypan blue. The plates were incubated at 28°C for 2 days, and the cellulolytic clones were detected by clear halos around the colonies [16]. Three clones were finally chosen based on their relatively higher cellulolytic activities among 309 cellulase-positive clones that showed good colonial development and visible clearing zones and were maintained on CMC agar.

### 2.3. Identification of Bacterial Isolates

The isolates were morphologically and physiologically characterized and identified up to genus level according to Bergey's Manual of Determinative Bacteriology (8th edition). An API 50 CHB kit (BioMérieux, Lyon, France) was also used for the identification of Gram-positive bacteria. API strips were inoculated with 24 h-grown cultures and then incubated at 28°C. The results were read according to the manufacturer's instructions and compared with other known *Bacillus subtilis* strains obtained from KACC and KCTC. Standard procedures [17] were used to analyze the clones for motility, sporulation, catalase, and Gram reaction.

### 2.4. Analyses of 16S rRNA Gene Sequences

Genes of 16S rRNA were sequenced and compared for identification of the bacterial isolates. The bacterial cells grown on CMC agar were harvested and used for chromosomal DNA isolation according to the protocols [18]. The chromosomal DNA was used as a template for amplification of 16S rRNA via the polymerase chain reaction (PCR). The primers used were 27F: 5′-AGAGTTTGATCATGGCTCAG-3′ as a forward primer and 1522 r: 5′-AAGGAGGTGATCCARCCGCA-3′ as a reverse primer. The PCR reaction mixture was composed of 5 *μ*L of template (50 ng/*μ*L), 5 *μ*L of 10x reaction buffer (100 mM Tris-HCl, 400 mM KCl, 500 *μ*g/mL bovine serum albumin, pH 8.3), 5 *μ*L of deoxynucleoside triphosphates (2.5 mM each), 1 *μ*L of each primer (10 pmol/*μ*L), 0.5 *μ*L of Taq DNA polymerase, and distilled sterile water to make final total volume of 50 *μ*L. The reaction mixture was incubated in a thermocycler (GMI, Ramsey, Minnesota, USA) programmed to run 30 cycles repeatedly (1 min at 94°C for denaturation, 1 min at 55°C for annealing, and 1.5 min at 72°C for polymerization) and, finally, further incubated at 72°C for 10 min for DNA amplification. The molecular sizes of the resulting PCR products were analyzed on 1.0% agarose gel to confirm 1.5 kb 16S rRNA. This 16S rRNA was purified using a DNA purification kit (QIAGEN, Valencia, California, USA), and its nucleotide sequences were determined by the dideoxy chain-termination method [19] using a BigDye Terminator v3.0 Sequencing Kit (Amersham Pharmacia Biotech, Piscataway, New Jersey, USA). The 16S rDNA sequences were confirmed and compared through a BLAST nucleotide search provided by the National Center for Biotechnology Information (NCBI) GenBank (U.S. National Library of Medicine, Bethesda, Maryland, USA). The nucleotide sequence similarity of each isolate was obtained using the Gendoc program. These sequence data have been submitted to the GenBank databases under accession no. HQ236379 for SL9-9 isolate, HQ236380 for C5-16 isolate, and HQ236381 for S52-2 isolate.

### 2.5. Preparation of Cellulolytic Enzyme Solutions

Starter cultures were prepared by transferring cells with an inoculation loop from the CMC agar plates to 100 mL of CMC liquid medium, the initial pH of which was adjusted to 7.0 if not specified, in 500 mL Erlenmeyer flasks. Two days after shaking incubation at 28°C, aliquots of 2 mL starter cultures were seeded into 200 mL of CMC liquid medium in 500 mL flasks. The flasks were further incubated on a shaker at 150 rpm for 7 days at 28°C. Cell growth was monitored by measuring optical density at 600 nm. Culture samples were taken every 24 h during incubation, and their cell-free supernatants (CFSs) were obtained by centrifugation (10,000 ×g, 5 min) and analyzed for cellulolytic activities. Meanwhile, the precipitated cells were suspended, washed in 5 mL of 0.05 M phosphate buffer (pH 6.5), and disrupted by sonication (150 mA, 20 min). The resulting supernatant was removed after centrifuging (12,000 ×g, 30 min) the sonicated cell suspension at 4°C. The remaining cell debris (CD) was resuspended in 1 mL of 0.05 M phosphate buffer (pH 6.5) and assayed for cellulolytic enzyme activities.

### 2.6. Enzyme Activity Assay

CMCase activity was measured by incubating 0.2 mL of enzyme solution with 0.5 mL of 1% (w/v) carboxymethyl cellulose, prepared in 0.1 M sodium acetate buffer (pH 5.0), and 0.3 mL of 0.1 M sodium acetate buffer (pH 5.0) for 10 min at 50°C. The reducing sugars liberated were estimated by the 3,5-dinitrosalicylic acid (DNS) method [20]. The enzyme reaction was stopped by the addition of 3 mL DNS reagent (dinitrosalicylic acid 1 g, NaOH, 16 g, potassium sodium tartarate 300 g, and distilled water up to 1 L) to the above 1 mL reaction mixture, boiled in capped glass tubes for 5 min, and cooled in cold water, and then optical density was measured at 540 nm. The CMCase activity was determined using a calibration curve for D-glucose. One unit of CMCase activity was defined as the amount of enzyme that released 1 *μ*mol of reducing sugars as glucose equivalents min^−1^.

Avicelase activity was measured by incubating 0.5 mL of enzyme solution with 1 g of Avicel, a microcrystalline cellulose, as substrate and 1.5 mL of 0.1 M sodium acetate buffer (pH 5.0) for 1 h at 50°C. After incubation, the reaction mixture was centrifuged at 10,000 ×g for 5 min, and then 1 mL of the supernatant was taken to determine reducing sugars by the DNS method. One unit of Avicelase activity was defined as the amount of enzyme that released 1 *μ*mol of reducing sugars as glucose equivalents min^−1^. 

Filter paper-hydrolytic (FPase) activity was measured by a procedure [21] in which Whatman no. 1 filter paper was used as a substrate. Fifty milligrams of the substrate suspended in 1.5 mL of 0.1 M sodium acetate buffer (pH 5.0) was incubated with 0.5 mL of enzyme solution at 50°C for 2 h. After incubation, the reaction mixture was centrifuged at 10,000 ×g for 5 min, and then 1 mL of the supernatant was taken for the determination of reducing sugars by the DNS method. One unit of FPase activity was defined as the amount of enzyme that released 1 *μ*mol of reducing sugars as glucose equivalents min^−1^.

*β*-glucosidase (or cellobiase) activity was measured by using *ρ*-nitrophenyl-*β*-D-glucopyranoside (pNPG) as a substrate. The hydrolysis of pNPG releases *ρ*-nitrophenol, a pigmented substance that can be measured spectrophotometrically at 400 nm. The reaction mixture, containing 0.5 mL of 1 mg/mL pNPG, 0.5 mL of 0.05 M sodium acetate buffer (pH 5.0), and 0.5 mL of enzyme solution, was incubated at 50°C for 1 h. The enzyme reaction was stopped by adding 2 mL of 1 M Na_2_CO_3_, and the absorbance was measured at 400 nm. One unit of *β*-glucosidase activity was defined as the amount of enzyme that released 1 *μ*mol of para-nitrophenol min^−1^.

Xylanase activity was measured by using Beechwood xylan as a substrate [22]. The reaction mixture containing 0.2 mL of crude enzyme, 0.5 mL of 1% xylan solution in 0.05M phosphate buffer (pH 6.0), and 0.3 mL of buffer (pH 6.0) was incubated at 50°C for 10 min. The enzymatic reaction was stopped by adding 3 mL of DNS reagent, boiled in capped glass tubes for 5 min, and cooled in cold water for color stabilization. The resulting optical density was measured at 520 nm. D-xylose was used as a standard for the preparation of a calibration curve. One unit of xylanase activity was defined as the amount of enzyme that released 1 *μ*mol of reducing sugars as xylose equivalents min^−1^.

## 3. Results and Discussion

### 3.1. Screening of Cellulolytic Bacteria

**Figure 1:**
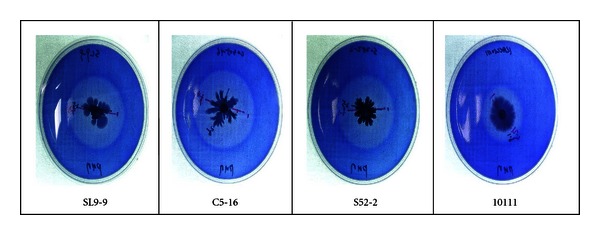
Bacterial cell growth and CMCase activity on CMC agar plates containing trypan blue. SL9-9, isolate from animal waste slurry; C5-16, isolate from compost; S52-2, isolate from soil; 10111, *B. subtilis *KACC10111. Clear halos resulting from cellulolytic activities could be detected around the colonies.

**Figure 2:**
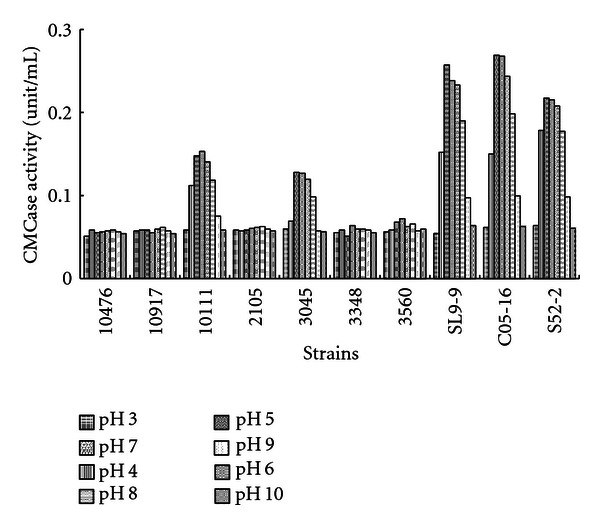
Comparison of carboxymethyl cellulase activity between *Bacillus* species at various cultivation pH. 10476, *B. licheniformis* KACC10476; 10917, *B. pumilus* KACC10917; 10111, *B. subtilis* KACC10111; 2105, *B. amylolicheniformis* KCTC2105; 3045, *B. licheniformis* KCTC3045; 3348, *B. pumilus* KCTC3348; 3560, *B. subtilis* KCTC3560; SL9-9, isolate from animal waste slurry; C5-16, isolate from compost; S52-2, isolate from soil. Bacterial cells were grown in carboxymethyl cellulose (CMC) media with various initial pH at 28°C for 3 days in a shaking incubator, and then their CMCase activities in cell-free culture supernatants were measured.

Cellulolytic bacteria were sought among 176 different samples collected from various environments such as soil, compost, and animal waste slurry on Jeju Island. Appropriate dilutions of each sample were placed on CMC agar plates. Positive clones showing good colonial development and a visible clearing zone were transferred to fresh CMC plates. A total of 309 positive clones were thus selected in the first round of screening. Their cellulolytic activities were confirmed by the trypan blue-staining method on CMC agar medium (Figure 1) and also by CMCase activity assay using cell-free supernatant obtained from the liquid cultures. The CMCase activities were examined and compared with those of other known *Bacillus species* obtained from KACC and KCTC. Finally, three clones showing relatively higher cellulolytic activity and broader pH optimum were selected (Figure 2). Their CMCase activities remained quite high around pH 5–8, and the isolates were designated as SL9-9, C5-16, and S52-2, from the animal waste slurry, compost, and soil, respectively. 

### 3.2. Identification of Isolated Bacteria

Morphological and cultural studies revealed that all the clones were Gram-positive and rod-shaped bacteria (Table 1). They were also catalase-positive, aerobic, moderate thermophiles. Their biochemical properties were further examined with an API 50CHB kit and compared with other *Bacillus subtilis* strains, namely, *B. subtilis* KACC10111 and *B. subtilis* KCTC3560 (Table 2). The three bacterial isolates showed slight differences from each other in such biochemical properties as methyl-*α*-D-glucopyranoside, amygdalin, salicin, D-maltose, D-lactose, inulin, glycogen, gentiobiose, and D-turanose utilization.

These three bacterial isolates were finally identified by 16S rRNA gene sequence analysis. Their sequences were entered into the nucleotide-nucleotide BLAST (NCBI) system, and percentage identities were established. The highest identity for the isolate SL9-9 (accession no. HQ236379) was 99% with the *Bacillus subtilis* strain BFAS (accession no. AY775778.1). The isolates C5-16 (accession no. HQ236380) and S52-2 (accession no. HQ236381) showed the highest identity at 99% with *Bacillus subtilis* strain CM19 (accession no. EU660332.1) and at 100% with *Bacillus subtilis* isolate C9-1 (accession no. EU257446.1), respectively. Based on their morphological, physiological, and genetic data, the three bacterial isolates were designated as *Bacillus subtilis* SL9-9, C5-16, and S52-2, respectively.

### 3.3. Production of Cellulolytic Enzymes by Bacterial Isolates

The three isolates were examined for CMCase, Avicelase, *β*-glucosidase, FPase, and xylanase production after cultivation in 200 mL of CMC liquid medium. *Bacillus subtilis* KACC10111, which showed higher CMCase activity than the other 6 *Bacillus species* obtained from KACC and KCTC (Figure 2), was used as a reference for enzyme activity comparisons.

**Figure 3:**
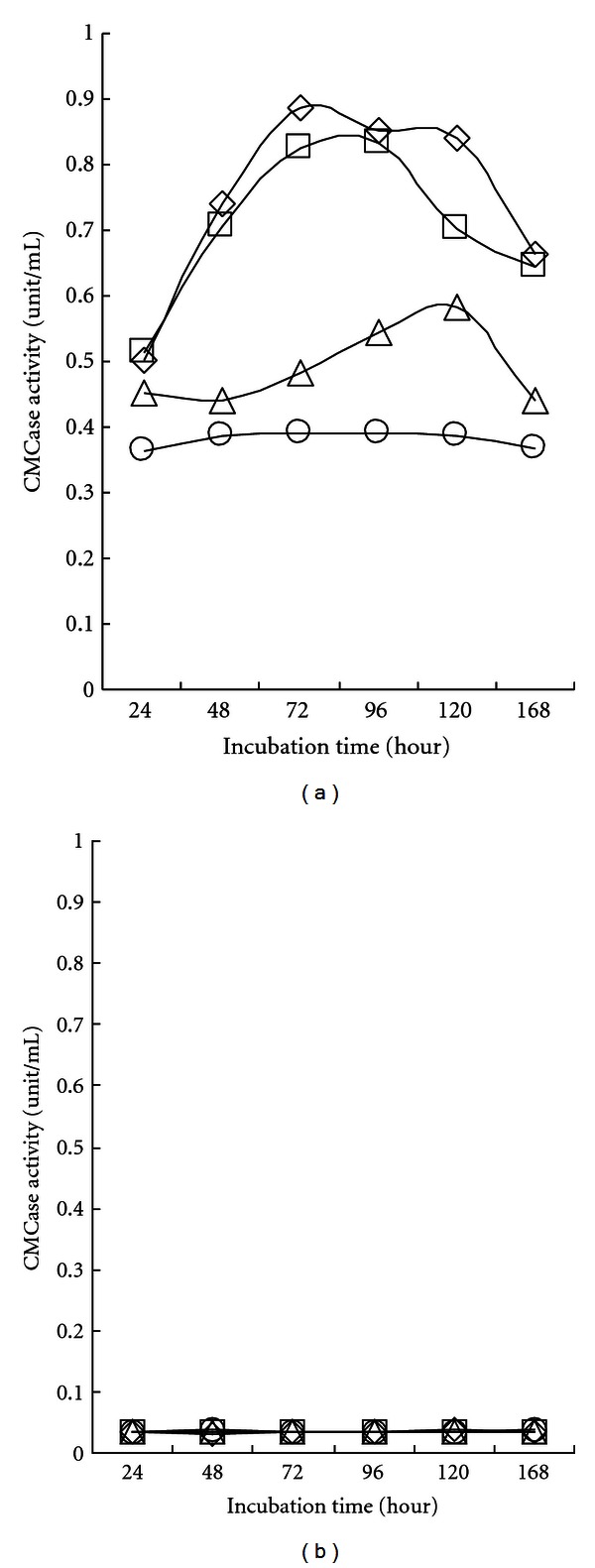
Carboxymethyl cellulase activity in cell-free culture supernatant (a) and cell debris (b) of isolated *Bacillus subtilis* strains. Bacterial cells (open rhombus, SL9-9; open square, C5-16; open triangle, S52-2; open circle, KACC10111) were grown in carboxymethyl cellulose (CMC) liquid medium (pH 7.0) on a shaker at 150 rpm for 7 days at 28°C. All cultures entered stationary phase in 5 days. Both cell-free culture supernatants (CFSs) and cell debris (CD) were assayed for CMCase activity. The mean values obtained from triplicate experiment were used to present results.

Figure 3 shows the CMCase activity profiles obtained during shaking incubation for 7 days with 10 g/L of carboxymethylcellulose as a carbon source. In the cell-free supernatant, both strains of SL9-9 and C5-16 showed considerable CMCase activity, reaching their maxima after 72 h of cultivation (0.9 and 0.8 unit/mL, resp.), while the other two strains, S52-2 and KACC10111, presented relatively lower activities. The CMCase activities decreased slightly after 120 h of cultivation. Some differences in endo-*β*-1,4-glucanase regulation among cellulolytic *Bacillus species* become apparent if one examines the timing of enzyme synthesis within a culture life cycle. There have been reports of cellulolytic enzyme synthesis during exponential growth [23] and after exponential growth [9, 10]. In the cell debris fraction, there was no observable CMCase activity (Figure 3(b)). Thus, CMCase was suggested as an extracellular enzyme.

**Figure 4:**
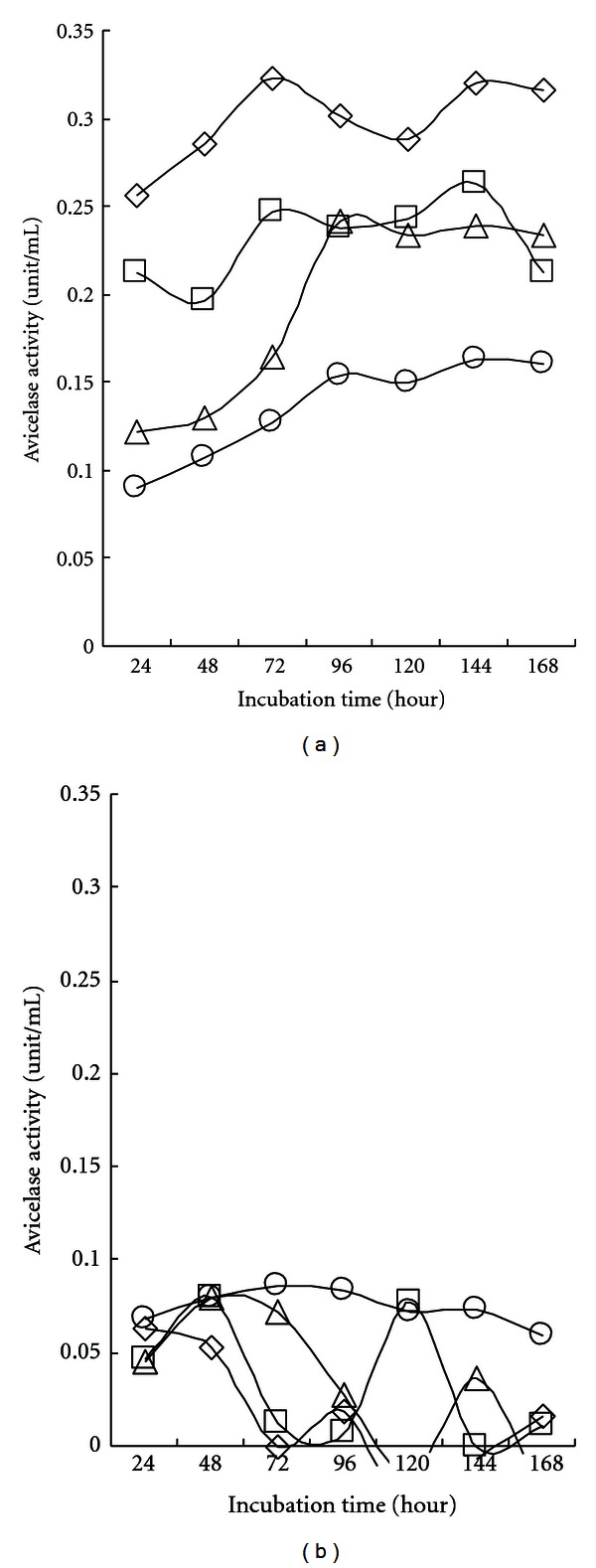
Avicelase activity in cell-free culture supernatant (a) and cell debris (b) of isolated *Bacillus subtilis *strains. Bacterial cells (open rhombus, SL9-9; open square, C5-16; open triangle, S52-2; open circle, KACC10111) were grown in carboxymethyl cellulose (CMC) liquid medium (pH 7.0) on a shaker at 150 rpm for 7 days at 28°C. All cultures entered stationary phase in 5 days. Both cell-free culture supernatants (CFSs) and cell debris (CD) were assayed for Avicelase activity. The mean values obtained from triplicate experiment were used to present results.

Figure 4 shows the Avicelase activity profiles obtained during shaking incubation for 7 days with 10 g/L of carboxymethyl cellulose as a carbon source. In the cell-free supernatant, all the strains produced considerable Avicelase activity and maintained maximum activity after 72–96 h of cultivation, although C5-16 showed a slight drop after 144 h of cultivation. 

On a whole, SL9-9 presented higher activity than the other isolates from the beginning of cultivation. In the cell debris fraction, there was no definite Avicelase activity (Figure 4(b)). The profiles of Avicelase activity (Figure 4) were somewhat similar to those of CMCase activity (Figure 3). In addition, when the same *Bacillus* strains were examined for endo-*β*-1,4-glucanase activity using Whatman no. 1 filter paper as a substrate, low hydrolytic activity levels (0.025–0.030 unit/mL) were observed and slightly increased as cultivation continued like the Avicelase activity profiles (data not shown). It is highly possible that both the Avicelase and CMCase (endo-*β*-1,4-glucanase) activity resulted from the same enzyme protein. Our results show some contrast to a previous report [10] in which the endo-*β*-1,4-glucanase produced by *B. subtilis* DLG was not able to significantly degrade crystalline cellulosic substrates. Fukumori et al. [24] also reported that endo-*β*-1,4-glucanases from alkalophilic *B. subtilis* strains 1139 and N-4 were capable of hydrolysing CMC, but could not degrade Avicel significantly. Hamamoto et al. [25] suggested that a synergistic function of the NH_2_-terminus and COOH-terminus of the endoglucanase in *Clostridium cellulovorans* is essential for the hydrolysis of crystalline cellulose. These phenomena suggest that crystalline cellulose-hydrolyzing activity does not depend on the same catalytic site of endo-*β*-1,4-glucanase.

**Figure 5:**
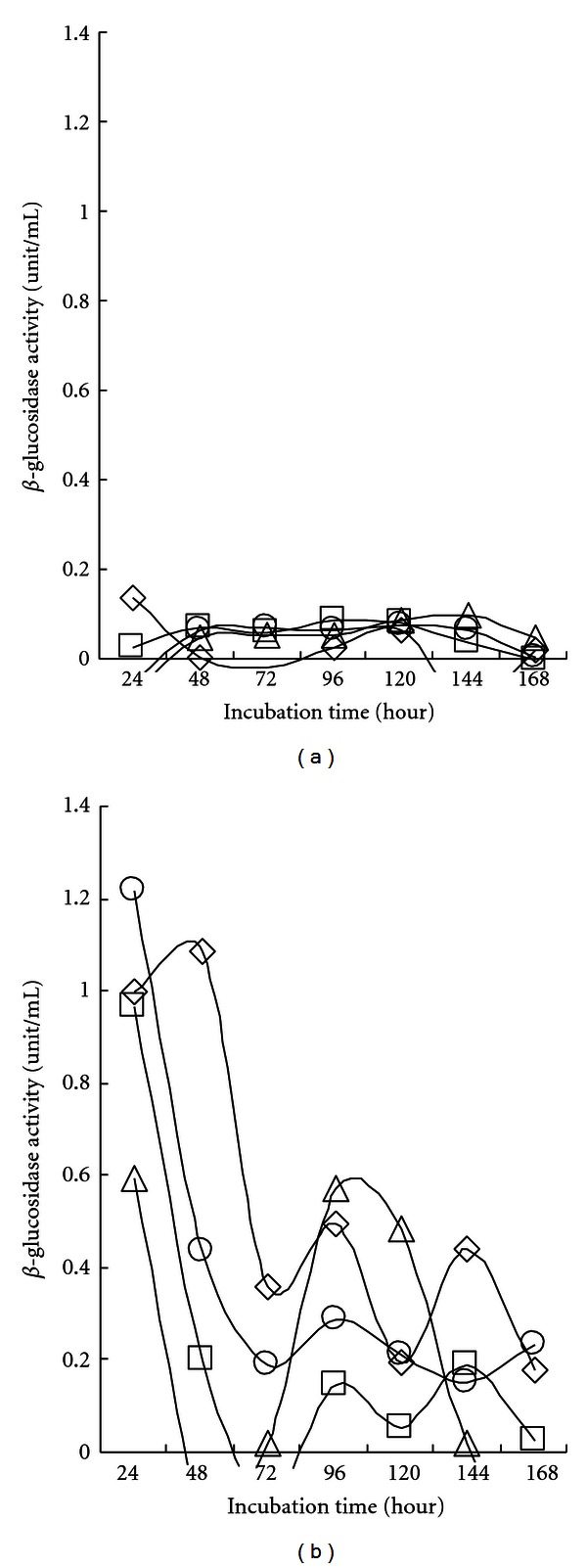
*β*-glucosidase activity in cell-free culture supernatant (a) and cell debris (b) of isolated *Bacillus subtilis *strains. Bacterial cells (open rhombus, SL9-9; open square, C5-16; open triangle, S52-2; open circle, KACC10111) were grown in carboxymethyl cellulose (CMC) liquid medium (pH 7.0) on a shaker at 150 rpm for 7 days at 28°C. All cultures entered stationary phase in 5 days. Both cell-free culture supernatants (CFSs) and cell debris (CD) were assayed for *β*-glucosidase activity. The mean values obtained from triplicate experiment were used to present results.

Figure 5 shows the *β*-glucosidase (or cellobiase) activity profiles obtained during shaking incubation for 7 days with 10 g/L of carboxymethylcellulose as a carbon source. In contrast to CMCase and Avicelase, no *β*-glucosidase activity was observed in the cell-free supernatant. However, all the strains showed considerable *β*-glucosidase activity in the cell debris fraction. Maximum activities (1.0, 1.0, 0.6, and 1.2 unit/mL for SL9-9 C5-16, S52-2, and KACC10111, resp.) were detected early after cultivation for 24 h. The enzyme activities subsequently decreased after reaching maximum values, and then second rises and falls were observed. A reason for the rise and fall in *β*-glucosidase activity might be the negative regulation of *β*-glucosidase gene expression by glucose level (catabolite repression) in the cells. *β*-glucosidase activities are inferred to be related to membrane-associated enzymes. So far, *β*-glucosidase has scarcely been reported in *Bacillus* strains, although its production by other bacteria like *Clostridium thermocellum *[26] and *Alcaligenes faecalis *[27] has been documented. Bartley et al. [28] reported that *β*-glucosidase in actinomycete *Microbispora bispora* was cell membrane bound. Pajni et al. [29] reported that all 34 strains of cellulolytic *Bacillus *species isolated from soil produced xylanase, and 82.4% of them also produced *β*-glucosidase. On the other hand, Dhillon et al. [11] found that *B. licheniformis* could grow in minimal media containing cellobiose, but failed to show the presence of cellobiase in either the cellular fraction or culture supernatant. It was hypothesized that the utilization of cellobiose even in the absence of cellobiase involved the enzyme cellobiose phosphorylase [10].

**Figure 6:**
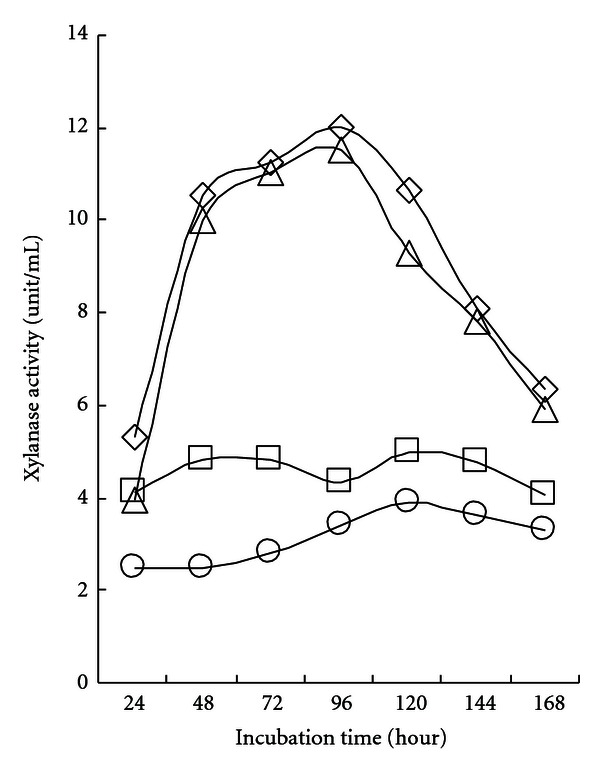
Xylanase activity in cell-free culture supernatant of isolated *Bacillus subtilis* strains. Bacterial cells (open rhombus, SL9-9; open square, C5-16; open triangle, S52-2; open circle, KACC10111) were grown in carboxymethyl cellulose (CMC) liquid medium (pH 7.0) on a shaker at 150 rpm for 7 days at 28°C. All cultures entered stationary phase in 5 days. Both cell-free culture supernatants (CFSs) and cell debris (CD) were assayed for xylanase activity. No xylanase activity was detected in the CD fraction. The mean values obtained from triplicate experiment were used to present results.

The *Bacillus* strains SL9-9 and S52-2 showed considerable xylanase activity in the cell-free culture supernatant, and their activities reached maximum values (12.0 and 11.5 unit/mL, resp.) after shaking culture for 96 h with 10 g/L of carboxymethylcellulose as a carbon source, as shown in Figure 6. The other two strains, C5-16 and KACC10111, presented lower overall activity from the beginning of cultivation. No xylanase activity was detected in the cell debris fraction. Xylanase production has been previously reported in *Bacillus *strains [30]. Pajni et al. [29] reported that all examined cellulolytic *Bacillus *species were also xylanase positive, and units of xylanase activity were found to be much higher as compared to corresponding CMCase activity units. Xylans, with a linear backbone of *β*-1,4-linked xylose residues, form the major group of hemicelluloses. Endoxylanases hydrolyze xylan to xylooligosaccharides and xylose residues, while *β*-xylosidases catalyze the release of xylosyl residues by the terminal attack of xylooligosaccharides. It is highly possible that the xylanase activities of our *Bacillus subtilis* strains came from the combined actions of independent endoxylanase and *β*-xylosidase enzymes.

Three cellulolytic bacterial strains, SL9-9, C5-16, and S52-2, were isolated and identified as *Bacillus subtilis* in this study. The isolates were not the same strains, showing slight differences in biochemical characteristics, 16S rRNA gene sequences, and production patterns of cellulases and xylanases. They had microcrystalline cellulose-hydrolytic activity in addition to *β*-glucosidase, hemicellulase, and endoglucanase activities. These strains are presently being employed in agriculture as a fertilizer supplement. They especially were quite effective as ingredients of an organic seedbed.

## Figures and Tables

**Table 1:** Morphological and physiological properties of the isolated cellulolytic bacteria.

Characteristics	Bacterial isolates		
	SL9-9	C5-16	S52-2
Gram staining	Positive (+)	Positive (+)	Positive (+)
Motility	+	+	+
Catalase	+	+	+
Cell shape	Rod	Rod	Rod
Size (L, *μ*m)	2.5~3.0	2.5~3.0	2.5~3.0
Colony appearance			
Shape	Round	Round	Round
Margin	Entire	Entire	Undulate
Elevation	Umbonate	Umbonate	Umbonate
Growth temp range (°C)^a^	15~50	15~55	15~50
Growth pH range^b^	5~10	4~9	5~9

^a^Incubated for 72 h; ^b^Incubated for 48 h.

**Table 2:** Biochemical properties of the isolated cellulolytic bacteria.

Test^a^		Strains			
				*B. subtilis *	*B. subtilis *
		SL9-9 C5-16 S52-2		KACC 10111	KCTC 3560
Control	−	−	−	−	−
Glycerol	**+**	**+**	**+**	**+**	**+**
Erythritol	−	−	−	−	−
L-arabinose	**+**	**+**	**+**	**+**	**+**
D-ribose	**+**	**+**	**+**	**+**	**+**
D-xylose	**+**	v	v	**+**	v
L-xylose	−	−	−	−	−
D-adonitol	−	−	−	−	−
D-glucose	**+**	**+**	**+**	**+**	**+**
D-fructose	**+**	**+**	**+**	**+**	**+**
D-mannose	**+**	**+**	**+**	**+**	**+**
L-sorbose	−	−	−	−	−
L-rhamnose	−	−	−	−	−
Dulcitol	−	−	−	−	−
Inositol	**+**	**+**	**+**	**+**	**+**
D-mannitol	**+**	**+**	**+**	**+**	**+**
D-sorbitol	**+**	**+**	**+**	**+**	**+**
Methyl-*α*D-glucopyranoside	**+**	**+**	−	**+**	**+**
N-acetyl-glucosamine	−	−	−	−	−
Amygdalin	**+**	**+**	−	**+**	v
Arbutin	**+**	**+**	**+**	**+**	**+**
Esculin ferric citrate	**+**	**+**	**+**	**+**	**+**
Methyl-*β*-xylopyranoside	−	−	−	−	−
D-lactose (bovine origin)	**+**	−	**+**	−	−
D-arabinose	−	−	−	−	−
Salicin	**+**	**+**	−	**+**	**+**
D-cellobiose	**+**	**+**	**+**	**+**	**+**
D-maltose	**+**	**+**	−	**+**	**+**
D-melibiose	**+**	**+**	**+**	**+**	v
D-saccharose	**+**	**+**	**+**	**+**	**+**
D-trehalose	**+**	**+**	**+**	**+**	**+**
Inulin	−	**+**	−	**+**	v
D-melezitose	−	−	−	−	−
Glycogen	**+**	**+**	−	**+**	**+**
Xylitol	−	−	−	−	−
Gentiobiose	−	v	−	−	v
D-turanose	−	**+**	−	**+**	v
D-lyxose	−	−	−	−	−
D-tagatose	−	−	−	−	−
D-fucose	−	−	−	−	−
L-fucose	−	−	−	−	−
D-arabitol	−	−	−	−	−
Potassium gluconate	−	**+**	−	**+**	−
Potassium2-ketogluconate	−	−	−	−	−
D-galactose	−	−	−	−	−
D-raffinose	**+**	**+**	**+**	**+**	v
Potassium5-ketogluconate	−	−	−	−	−
Methyl-*α*D-mannopyranoside	−	−	−	−	−
AmiDon (Starch)	**+**	**+**	−	**+**	**+**
L-arabitol	−	−	−	−	−

^a^API 50CHB Kit (BioMérieux, France) was used to determine positive (+) or negative (−); v: variable.
